# Oral magnesium supplementation improves glycemic control in older Chinese adults with pre-diabetes and hypomagnesemia: a randomized controlled trial

**DOI:** 10.3389/fnut.2026.1765308

**Published:** 2026-02-11

**Authors:** Jingxin Yang, Huidi Zhang, Yuting Li, Wenxuan Wu, Min Pan, Jingjing Wang, Guoxun Li, Ying Wu, Chunlei Guo, Lichen Yang, Jing Ding, Gangqiang Ding

**Affiliations:** 1 NHC Key Laboratory of Public Nutrition and Health, National Institute for Nutrition and Health, Chinese Center for Disease Control and Prevention, Beijing, China; 2 Yuetan Community Health Center, Fuxing Hospital Capital Medical University of Medical Science, Beijing, China; 3 Department of Nutrition, The Third Central Hospital of Tianjin, Tianjin, China; 4 Guangwai Hospital, Beijing, China

**Keywords:** magnesium deficiency, magnesium supplement, older Chinese adults, pre-diabetes, RCT

## Abstract

**Purpose:**

Pre-diabetes significantly increases the risk of type 2 diabetes and cardiovascular disease. Magnesium deficiency is common and may contribute to dysglycemia. However, evidence for the efficacy of magnesium supplementation in pre-diabetes, especially in older adults with hypomagnesemia, remains limited and inconclusive. This exploratory trial aimed to evaluate the effects of oral magnesium supplementation on glycemic parameters and conducted exploratory metabolomic profiling in this population.

**Methods:**

In this 4-month, randomized, double-blind, placebo-controlled trial, 71 community-dwelling older adults (mean age 68.7 ± 6.0 years) with pre-diabetes (fasting plasma glucose ≥5.6 mmol/L and/or HbA1c 5.7%−6.5%) and hypomagnesemia (plasma magnesium ≤ 0.80 mmol/L) were enrolled. Participants were randomly assigned to receive either magnesium oxide (360 mg elemental Mg/day) or an identical placebo once daily. The primary outcome was the change in fasting plasma glucose (FPG). Secondary outcomes included changes in insulin, HOMA-IR, HbA1c, glycated albumin, and inflammatory markers (hs-CRP, IL-6). Exploratory non-targeted metabolomic profiling was performed. Data were analyzed using ANCOVA adjusted for baseline values, following the intention-to-treat principle.

**Results:**

Sixty-five participants completed the trial. At baseline, the magnesium group had significantly higher insulin and HOMA-IR levels (both *p* < 0.05); analyses were adjusted accordingly. Compared to placebo, magnesium supplementation significantly increased plasma magnesium (adjusted mean difference: 0.056 mmol/L, 95% CI: 0.028 to 0.085; *p* < 0.001) and reduced FPG (adjusted mean difference: −0.497 mmol/L, 95% CI: −0.818 to −0.176; *p* = 0.003). The reduction in HOMA-IR favored the magnesium group but was not statistically significant after adjustment (*p* = 0.296). No significant between-group differences were observed for HbA1c, insulin, C-peptide, glycated albumin, or inflammatory markers. Exploratory metabolomics revealed alterations in putatively identified metabolites, with pathway analysis suggesting involvement of lipid and insulin resistance-related pathways; these findings are considered hypothesis-generating.

**Conclusion:**

In older adults with pre-diabetes and hypomagnesemia, magnesium supplementation effectively corrected magnesium deficiency and reduced FPG, but did not improve other glycemic indices including HbA1c or insulin resistance. The clinical significance of the isolated FPG reduction remains uncertain. The metabolomic findings require validation. Larger, longer-term trials are needed to determine if magnesium supplementation can prevent diabetes in this population.

**Clinical trial registration:**

www.chictr.org.cn, identifier: ChiCTR2100047666.

## 1 Introduction

Pre-diabetes mellitus (Pre-DM), a high-risk glycemic state for progression to type 2 diabetes (T2DM) and cardiovascular disease, imposes a substantial global health burden (1). While this intermediate stage presents a critical window for intervention to reverse dysglycemia, effective and scalable strategies remain a priority (2–4).

Magnesium (Mg), an essential cofactor in numerous enzymes governing glucose metabolism and insulin signaling, is of particular interest (5). Substantial epidemiological evidence consistently links lower dietary magnesium intake and hypomagnesemia to an increased risk of both pre-diabetes and T2DM (6). A recent meta-analysis further confirmed that individuals with pre-diabetes have significantly lower serum magnesium levels compared to those with normal glucose tolerance, highlighting a consistent association (7). This association is mechanistically plausible, as magnesium is vital for insulin receptor activity and post-receptor signaling (8).

However, evidence from interventional trials is sparse and inconclusive (9), particularly for key populations such as older adults (10). To our knowledge, only two small randomized controlled trials (RCTs) have specifically examined magnesium supplementation in individuals with pre-diabetes, reporting mixed effects on glycemic control (11, 12). Notably, these studies did not account for baseline magnesium status or dietary intake, and neither focused on older adults—a demographic with a high prevalence of pre-diabetes, face age-related declines in nutrient absorption, and often contend with multimorbidity (13). A targeted intervention addressing magnesium deficiency in older adults with pre-diabetes could offer a simple nutritional strategy to support metabolic health.

Therefore, we conducted an exploratory, randomized, double-blind, placebo-controlled trial to investigate the effects of oral magnesium supplementation on glycemic parameters in older Chinese adults with pre-diabetes and concurrent hypomagnesemia. By integrating clinical biochemistry with exploratory metabolomics, this study aims to evaluate the potential of targeted magnesium repletion for improving metabolic markers in this at-risk population.

## 2 Materials and methods

### 2.1 Study design and participants

This 16-week, randomized, double-blind, placebo-controlled trial was conducted between October 2022 and November 2023 at community and two hospital sites. The study protocol was approved by the Ethics Committee of the National Institute for Nutrition and Health, Chinese Centre for Disease Control and Prevention (No. 2021-015) and has been made publicly accessible on the clinical trial registry website (ChiCTR2100047666). All pre-specified primary (change in fasting plasma glucose) and secondary outcomes (changes in insulin, HOMA-IR, HbA1c, glycated albumin, hs-CRP, and IL-6) were explicitly listed in the trial registry record. The non-targeted metabolomic profiling reported in this manuscript was a pre-planned exploratory analysis, as indicated in the registered protocol. The trial was conducted in accordance with the principles of the Declaration of Helsinki. All participants provided written informed consent.

**Figure 1:**
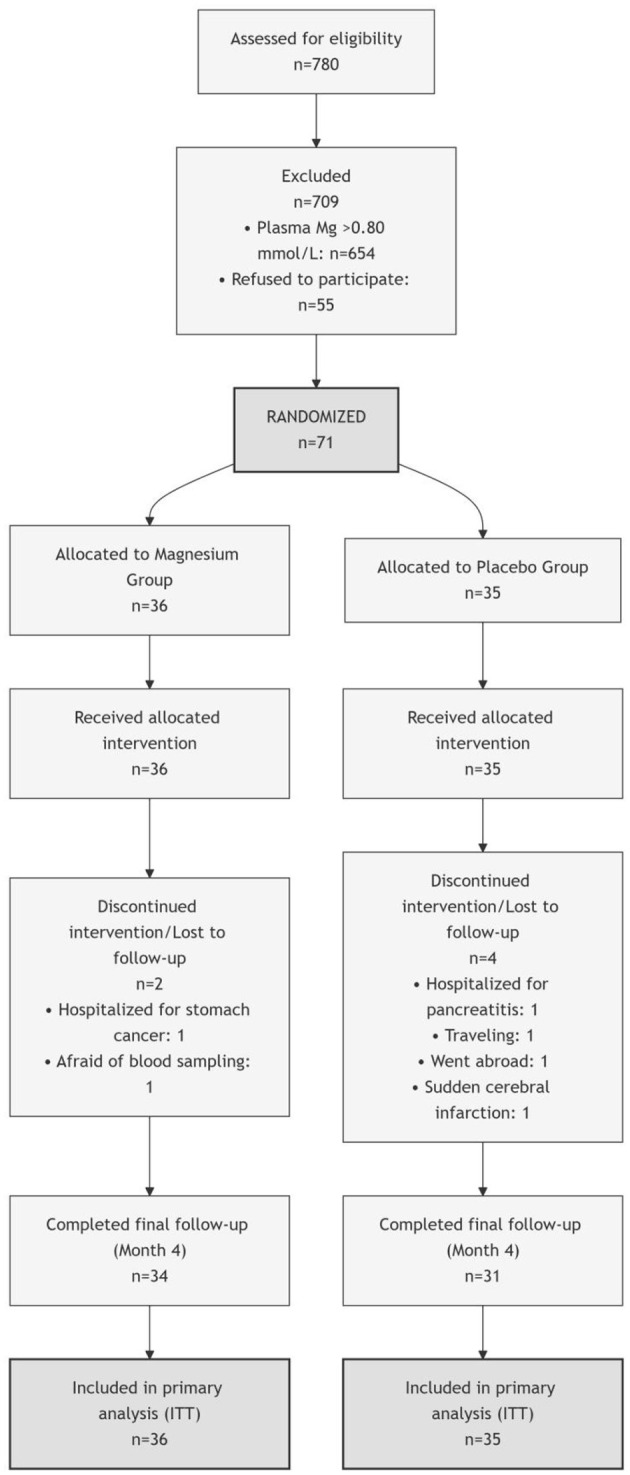
Consort flow diagram of the study participants.

We enrolled 71 community-dwelling adults with pre-diabetes, defined as fasting plasma glucose (FPG) ≥5.6 mmol/L and/or glycated haemoglobin (HbA1c) 5.7%−6.5% (14), and concurrent hypomagnesemia. Hypomagnesemia was defined as plasma Mg concentration ≤ 0.80 mmol/L, a cutoff consistent with clinical guidelines and previous studies in similar populations (15). A complete CONSORT flow diagram of participant screening, enrolment, allocation, follow-up, and analysis is provided in Figure 1. Detailed inclusion and exclusion criteria are summarized in Supplementary Table S1. Key exclusion criteria included: (1) diagnosis of diabetes mellitus; (2) current use of medications known to affect glucose or mineral metabolism (e.g., diuretics, insulin, oral hypoglycemic agents).

### 2.2 Randomization, allocation concealment, and blinding

Eligible participants were randomly assigned in a 1:1 ratio to either the magnesium supplement group or the placebo group. The randomization sequence was computer-generated by an independent statistician (W.WX) using block randomization (block size of four), stratified by age (< 65 vs. ≥65 years), sex, baseline plasma Mg concentration ( ≤ 0.70 vs. >0.70 mmol/L), and baseline FPG (< 6.1 vs. ≥6.1 mmol/L). Allocation concealment was ensured using sequentially numbered, opaque, sealed envelopes (SNOSE). The envelopes were prepared by the independent statistician and kept securely by the study pharmacist (P.M), who was not involved in participant recruitment or outcome assessment.

Participants, research staff (Y.JX) involved in participant management and outcome assessment, laboratory personnel, and data analysts were all blinded to group assignment throughout the trial. The magnesium oxide tablets (providing 360 mg of elemental magnesium per day) and the visually identical placebo tablets (composed primarily of starch) were manufactured, coded, and supplied by Amway Corporation. The blinding was maintained until all statistical analyses were completed.

### 2.3 Intervention and compliance

Participants were instructed to take two tablets (either magnesium or placebo) once daily with a meal for 16 weeks. Compliance was monitored at each monthly visit via pill counts of returned medication bottles and review of participant medication diaries. Adherence was calculated as (number of tablets taken/number of tablets prescribed) × 100%. Good compliance was defined as taking ≥80% of the prescribed tablets. Any adverse events or side effects, particularly gastrointestinal symptoms (e.g., diarrhoea, abdominal discomfort), were recorded at each follow-up visit using a structured questionnaire.

### 2.4 Data collection and measurements

Data were collected at baseline (Week 0), an interim visit (Month 2), and at the end of the intervention (Month 4). After a 10–12 hour overnight fast, venous blood samples were collected into appropriate vacutainers for plasma, serum, and whole blood separation. A spot urine sample was also collected.

Anthropometric measurements included body weight, height, waist circumference, and blood pressure (measured in triplicate after a 5-min rest). Dietary intake was assessed using a combination of tools to improve accuracy. At baseline, three consecutive days of 24-hour weighed dietary records were conducted, supplemented by a validated semi-quantitative food frequency questionnaire (FFQ) to assess habitual magnesium intake.

### 2.5 Biochemical analyze

Plasma magnesium concentration was determined by inductively coupled plasma mass spectrometry (ICP-MS). Glucose metabolism markers, including FPG, insulin, C-peptide, HbA1c (by HPLC), and glycated albumin, were measured. Homeostasis model assessment of insulin resistance (HOMA-IR) was calculated. HOMA-IR was used to measure the insulin resistance (IR) of hepatic and was calculated as insulin (μU/mL) × fasting plasma glucose (FPG, mmol/L)/22.5 (14). Inflammatory markers [high-sensitivity C-reactive protein (hs-CRP) and interleukin-6 (IL-6)] were assayed by standard clinical biochemical methods and electrochemical luminescence, respectively. Liver and kidney function tests were performed for safety monitoring.

### 2.6 Non-targeted metabolomic profiling

Plasma samples underwent non-targeted metabolomic analysis using ultra-high-performance liquid chromatography coupled with high-resolution mass spectrometry (UHPLC-MS). Chromatographic separation was performed on a Vanquish UHPLC system (Thermo Scientific, Massachusetts, USA), and mass spectrometric detection was conducted on a Q Exactive HF-X hybrid quadrupole-Orbitrap mass spectrometer (Thermo Scientific, Massachusetts, USA) in both positive and negative electrospray ionization modes. Metabolites were extracted with a methanol/acetonitrile solvent system. Data processing included peak alignment, identification, and normalization. Multivariate statistical analyses, including principal component analysis (PCA) and orthogonal partial least squares-discriminant analysis (OPLS-DA), were performed to assess group separation. Differential metabolites were initially screened using variable importance in projection (VIP) >1.0 from OPLS-DA and *p* < 0.05 from Student's *t* test. To control for false discoveries, we further applied a false discovery rate (FDR) correction (Benjamini–Hochberg method), with FDR < 0.05 as the significance threshold for differential abundance. Metabolite identification remained putative pending MS/MS validation. Pathway enrichment analysis was conducted using the Kyoto Encyclopaedia of Genes and Genomes (KEGG) database based on these putatively identified metabolites, and results are presented as exploratory and hypothesis-generating.

### 2.7 Statistical analysis

The primary efficacy endpoint was the change from baseline to Month 4 in FPG. Secondary endpoints included changes in insulin, HOMA-IR, HbA1c, glycated albumin, hs-CRP, and IL-6. Analyses were performed following the intention-to-treat (ITT) principle, including all randomized participants (*n* = 71). For the primary and continuous secondary outcomes, missing data at Month 4 were imputed using the last observation carried forward (LOCF) method as a primary approach. A sensitivity analysis using complete cases only was also performed.

Baseline characteristics were compared between groups using independent samples *t*-tests or Mann–Whitney *U*-tests for continuous variables and chi-square or Fisher's exact tests for categorical variables. For the primary and key secondary outcomes, between-group differences in the change from baseline to Month 4 were analyzed using analysis of covariance (ANCOVA), adjusting for the respective baseline value as a covariate. Model assumptions (normality of residuals and homoscedasticity) were assessed using Shapiro–Wilk and Levene's tests, respectively; no substantial violations were observed. Results are presented as adjusted mean differences with 95% confidence intervals (CIs). Within-group changes from baseline were assessed using paired *t*-tests.

For the exploratory non-targeted metabolomics analysis, orthogonal partial least squares-discriminant analysis (OPLS-DA) was used to visualize group separation. Model validity was evaluated using a permutation test (*n* = 200) with the Q^2^ intercept *p*-value < 0.05 indicating robustness against overfitting. Differential metabolites were identified using variable importance in projection (VIP) >1.0 from OPLS-DA and false discovery rate (FDR)-corrected *p* < 0.05 (Benjamini–Hochberg method) to control for multiple comparisons across metabolites. Pathway enrichment analysis was conducted using the Kyoto Encyclopaedia of Genes and Genomes (KEGG) database and is presented as hypothesis-generating and descriptive in nature.

All reported *p* values are two-sided. A *p* value < 0.05 was considered statistically significant for primary and pre-specified secondary outcomes. Given the exploratory nature of the metabolomics analysis and the multiplicity of secondary/exploratory outcomes, findings from these analyses should be interpreted as hypothesis-generating. Statistical analyses were performed using SPSS Statistics (version 26.0, IBM Corp.) and R (version 4.3.0).

### 2.8 Sample size calculation

The sample size was calculated based on the primary endpoint (change in FPG). Based on a previous artical of magnesium supplementation in individuals with pre-diabetes (11), we assumed a between-group difference in FPG change of 0.66 mmol/L (SD = 0.65 mmol/L). To achieve 90% power with a two-sided alpha of 0.05, 25 participants per group were required. Anticipating a 20% dropout rate over 16 weeks, we aimed to recruit at least 30 participants per group, resulting in a final target sample size of 71 randomized participants.

### 2.9 Safety monitoring

All adverse events (AEs) were recorded throughout the study. The incidence of gastrointestinal AEs (e.g., diarrhoea, nausea, abdominal pain), a known side effect of magnesium oxide, was specifically monitored and compared between groups.

## 3 Results

### 3.1 Study population and baseline characteristics

Of the 780 individuals initially screened, 71 eligible participants with pre-diabetes and hypomagnesemia were enrolled and randomized. During the 16-week intervention, six participants withdrew for personal reasons, resulting in 65 completers (34 in the Mg group, 31 in the placebo group). Overall compliance was 91.6%. The flow of participants is detailed in Figure 1. The intervention was well-tolerated, with no intervention-related adverse events reported in either group. All participant discontinuations were for unrelated reasons (Figure 1).

Baseline characteristics are presented in Table 1. While demographic variables (age, BMI, waist circumference, sex, and smoking status) were comparable between groups, significant baseline imbalances were noted for two metabolic parameters, including insulin (12.0 ± 7.7 vs. 16.6 ± 10.7 μIU/ml, *p* = 0.040) and HOMA-IR (3.3 ± 2.2 vs. 5.4 ± 5.1, *p* = 0.034). All subsequent analyses were adjusted for these baseline differences as detailed in the statistical methods.

**Table 1:** Baseline characteristics of study participants.

**Variable**	**Placebo group (*n* = 35)**	**Mg supplemented group (*n* = 36)**	***p* value**
Age, years	69.4 ± 4.5	68.0 ± 7.2	0.327
BMI, kg/m^2^	24.6 ± 2.7	24.8 ± 3.4	0.791
Waist, cm	83.7 ± 8.0	83.0 ± 7.6	0.711
Male, *n* (%)	21 (60.0%)	13 (36.1%)	0.076
Smoking, *n* (%)	8 (22.9%)	5 (13.9%)	0.503
Mg, mmol/L	0.78 ± 0.05	0.78 ± 0.05	0.809
FPG, mmol/L	6.17 ± 0.68	6.69 ± 1.84	0.118
HbA1c, %	5.79 ± 0.42	5.99 ± 0.72	0.143
Insulin, μIU/ml	12.0 ± 7.7	16.6 ± 10.7	0.040
HOMA_IR	3.3 ± 2.2	5.4 ± 5.1	0.034
C_peptide, ng/ml	2.64 ± 1.07	2.65 ± 1.07	0.969
GA, %	14.89 ± 2.76	14.77 ± 3.34	0.864
IL6, pg/ml	3.3 ± 3.7	2.8 ± 3.2	0.495
HsCRP, mg/L	2.1 ± 2.7	1.8 ± 1.6	0.492

BMI, body mass index; Mg, magnesium; FPG, fasting plasma glucose; HbA1c, glycosylated hemoglobin; GA, glycated albumin; IL-6, interleukin-6; Hs-CRP, highly sensitive C-reactive protein. Bold values indicate significant (*p* < 0.05).

### 3.2 Dietary intake

**Figure 2:**
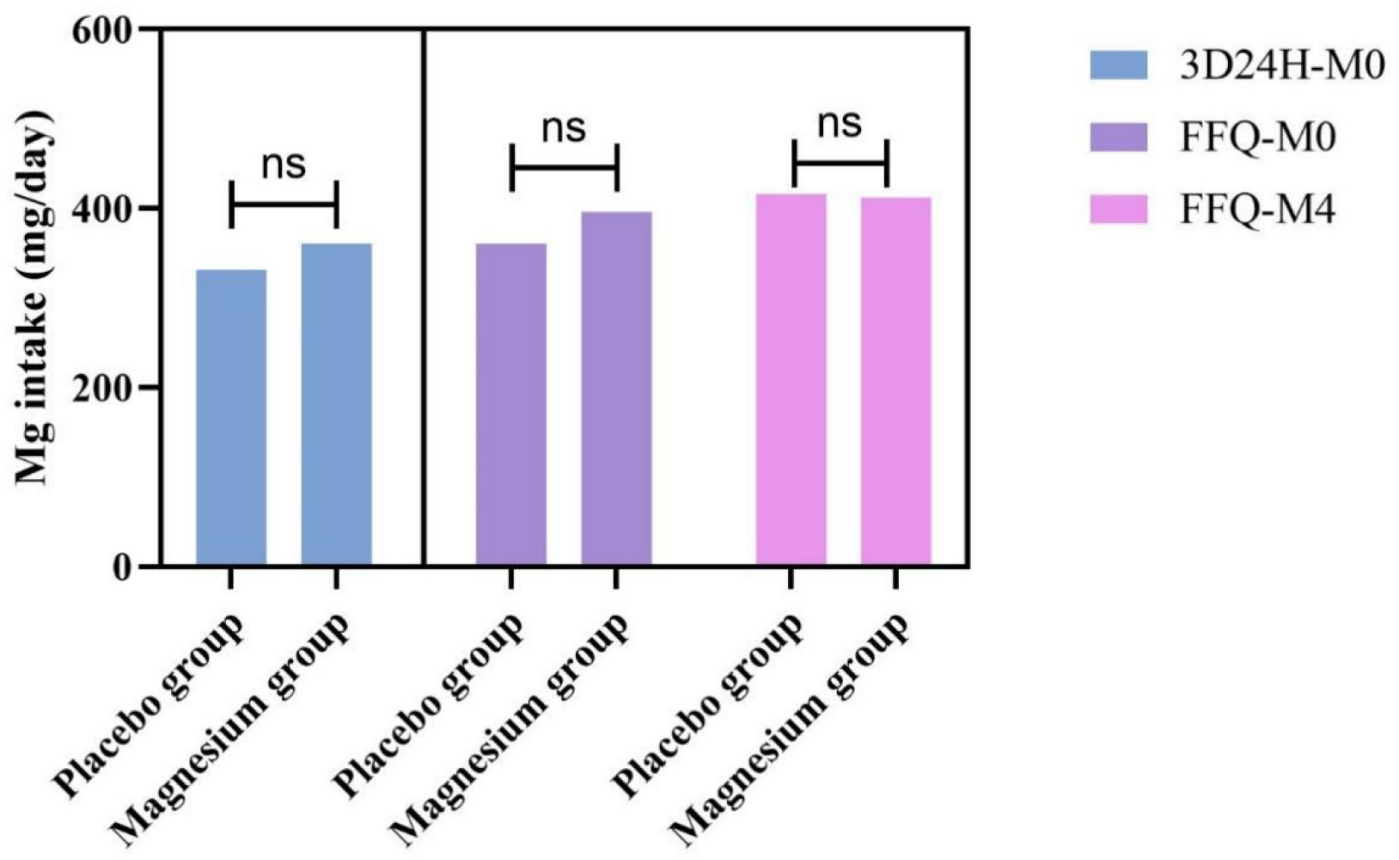
Comparison of dietary magnesium intake between magnesium supplement group and placebo control group in different dietary survey methods at different periods.

Dietary intake at baseline, assessed by both a three-day 24-hour dietary weighed and a food frequency questionnaire (FFQ), showed no significant differences between groups in energy, macronutrients, or dietary magnesium intake (Table 2). Dietary calcium intake assessed by FFQ was higher in the magnesium group at baseline (*p* = 0.049). Dietary intake remained stable throughout the study, with no significant between-group difference in magnesium intake at the end of the intervention (*p* = 0.899), as illustrated in Figure 2.

**Table 2:** Comparison of the results of a three-day 24-hour dietary survey and food frequency questionnaire (FFQ).

**Variable**	3D24H-M0 (***n*** = 64)			FFQ-M0 (***n*** = 71)		
	**Placebo group (*n* = 32)**	**Mg supplemented group (*n* = 32)**	***p-*value**	**Placebo group (*n* = 35)**	**Mg supplemented group (*n* = 36)**	***p-*value**
Energy (kcal)	1,870.23 ± 653.08	1,926.04 ± 665.59	0.736	1,726.30 ± 472.68	1,739.13 ± 575.90	0.919
Protein (g)	75.34 ± 27.92	79.77 ± 25.18	0.508	71.36 ± 23.87	78.08 ± 24.33	0.244
Fat (g)	67.58 ± 38.79	69.99 ± 33.68	0.792	50.54 ± 21.10	50.20 ± 17.42	0.941
CHO (g)	254.44 ± 88.60	258.09 ± 105.71	0.882	246.39 ± 90.38	250.31 ± 106.86	0.868
Calcium (mg)	621.90 ± 257.34	669.41 ± 323.85	0.518	675.70 ± 267.54	836.55 ± 395.66	**0.049**
Mg (mg)	343.04 ± 133.20	366.18 ± 146.55	0.508	360.75 ± 140.03	396.12 ± 174.48	0.350

3D24H, three-day 24-hour dietary survey; FFQ, food frequency questionnaire; M0, month 0; Mg, magnesium; CHO, Carbohydrate.

### 3.3 Effects of magnesium supplementation on glycemic and metabolic parameters

Changes in primary and secondary biochemical outcomes from baseline to 4 months are summarized in Table 3 and detailed in Supplementary Table S2. The primary analysis using ANCOVA, adjusted for baseline values, sex, insulin, and HOMA-IR, showed that magnesium supplementation significantly increased serum magnesium levels compared to placebo (effect size: 0.056 mmol/L, 95% CI: 0.028 to 0.085; *p* < 0.001).

**Table 3:** Intervention effects on biochemical outcomes (mean ± SD).

**Variable**	**Placebo_Δ**	**Mg_Δ**	**Δ_*p*-value**	**ANCOVA *p*-value**	**Effect size (95% CI)**
Mg, mmol/L	0.040 ± 0.059	0.112 ± 0.076	< 0.001	< 0.001	0.056 (0.028 to 0.085)
FPG, mmol/L	0.225 ± 0.587	−0.424 ± 0.979	0.001	0.003	−0.497 (−0.818 to −0.176)
HbA1c, %	0.055 ± 0.289	−0.114 ± 0.523	0.096	0.203	−0.109 (−0.274 to 0.057)
Insulin, μIU/mL	0.143 ± 4.484	−1.613 ± 6.541	0.191	0.581	−0.739 (−3.346 to 1.869)
HOMA_IR	0.242 ± 1.662	−0.879 ± 2.736	0.041	0.296	−0.495 (−1.417 to 0.426)
C_peptide, ng/ml	−0.112 ± 0.429	0.291 ± 1.248	0.074	0.512	0.126 (−0.248 to 0.499)
GA, %	−1.439 ± 2.030	−1.392 ± 2.635	0.934	0.983	0.007 (−0.611 to 0.625)
IL6, pg/ml	−0.827 ± 3.523	0.396 ± 1.990	0.079	0.116	0.835 (−0.192 to 1.863)
HsCRP, mg/L	−0.319 ± 2.888	−0.339 ± 1.936	0.972	0.457	−0.361 (−1.307 to 0.584)

Values represent mean change from baseline ± standard deviation; Δ represents the change from baseline; ANCOVA analysis adjusted for baseline values, sex, insulin, and HOMA-IR. Mg, magnesium; FPG, fasting plasma glucose; HbA1c, glycosylated hemoglobin; GA, glycated albumin; IL-6, interleukin-6; Hs-CRP, highly sensitive C-reactive protein.

**Figure 3:**
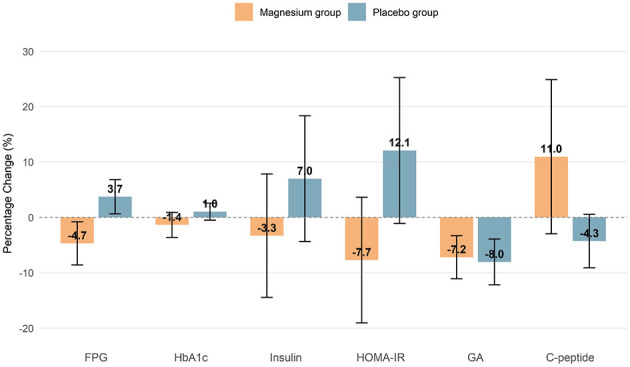
Comparison of percentage changes in glucose metabolism indexes between the placebo and magnesium groups. Data are presented as mean with 95% confidence intervals (error bars). The percentage change was calculated as (M4–M0)/M0 × 100%. FPG, fasting plasma glucose; GA, glycated albumin; HOMA-IR, homeostatic model assessment of insulin resistance. Extreme values for C-peptide (outside Q1–2 × IQR to Q3 + 2 × IQR) were excluded from analysis.

Regarding glycemic parameters, magnesium supplementation led to a significant reduction in fasting plasma glucose (FPG) compared to placebo (effect size: −0.497 mmol/L, 95% CI: −0.818 to −0.176; *p* = 0.003). The change in HOMA-IR favored the magnesium group but was not statistically significant in the adjusted model (effect size: −0.495, 95% CI: −1.417 to 0.426; *p* = 0.296). After applying False Discovery Rate (FDR) correction for multiple comparisons (Supplementary Table S3), the between-group difference in FPG remained significant (*q* < 0.05). No statistically significant between-group differences were observed for HbA1c, insulin, C-peptide, glycated albumin (GA), interleukin-6 (IL-6), or high-sensitivity C-reactive protein (hs-CRP) in either the primary or FDR-corrected analyses (Table 3; Supplementary Table S3). The percentage changes in key glucose metabolism indices are visually compared in Figure 3.

### 3.4 Exploratory non-targeted metabolomic profiling

Serum samples from the magnesium group at baseline (M0) and 4 months (M4) underwent exploratory non-targeted metabolomic analysis. Quality control included pooled QC samples and internal standards for normalization. In PCA, QC samples clustered tightly and separately from experimental samples, indicating analytical stability (Supplementary Figure S1).

**Figure 4:**
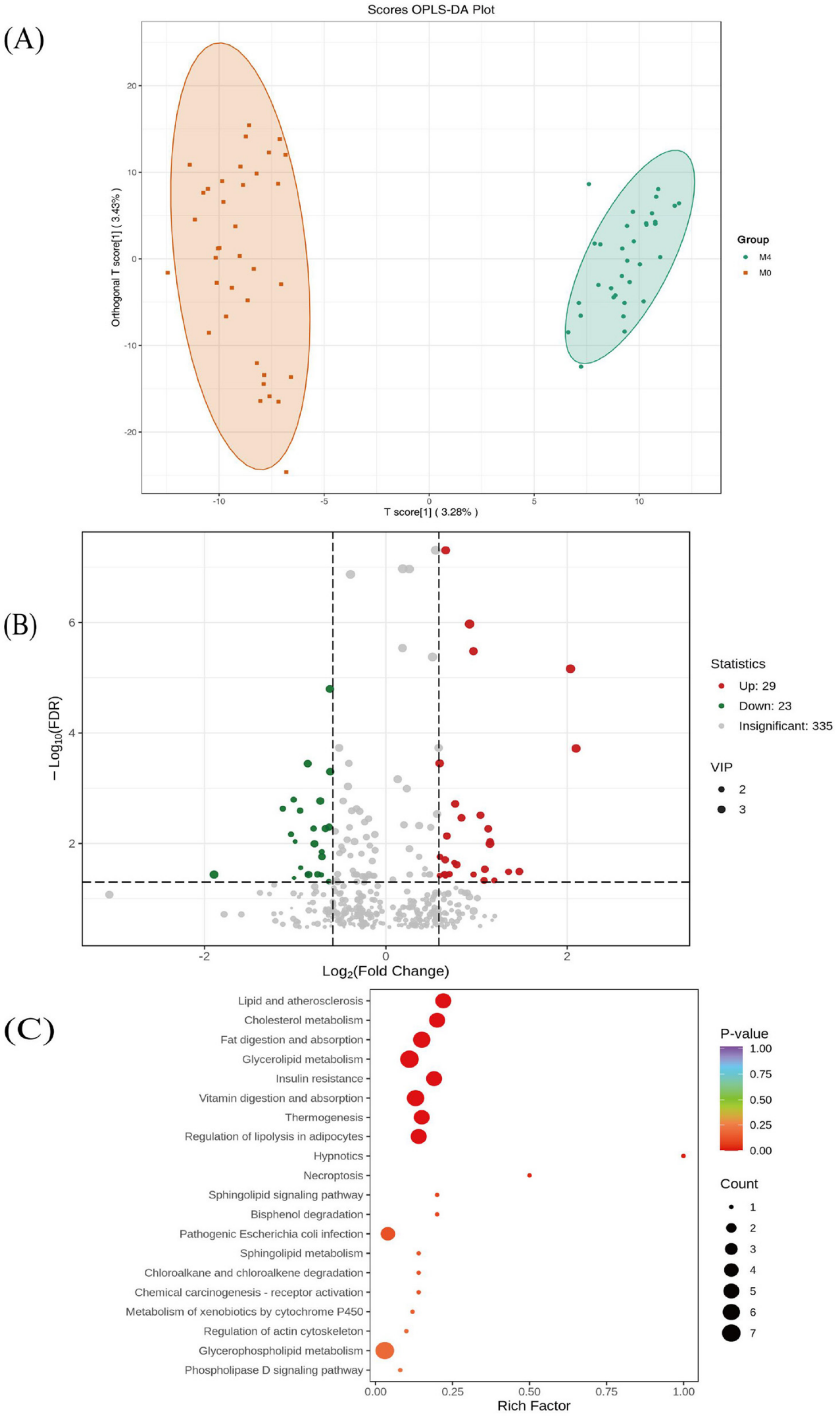
Metabolomic analysis of plasma samples after magnesium intervention. **(A)** OPLS-DA score plot comparing metabolic profiles of the magnesium (Mg) at baseline (M0) and after 4 months (M4). The horizontal axis represents the predicted principal component, illustrating the differences between groups along this direction; the vertical axis represents the orthogonal principal component, indicating the variability within groups along this direction. The percentage indicates the contribution of each component to the dataset explanation. Each point in the graph represents a sample, with samples from the same group displayed in the same color, and “Group” denotes the grouping. **(B)** Volcano plot of differential metabolites at M4. The x-axis is log_2_ (fold change, M4/M0), and the y-axis is –log_10_ (*p*-value) from *t*-tests. The horizontal axis shows the log2-transformed fold change (log2FC) in the relative content of each metabolite between two sample groups. The larger the absolute value on the horizontal axis, the greater the difference in relative content of that substance between the two groups. Under the screening conditions of VIP + FC + *p*-value: the vertical axis indicates the level of differential significance (–log10*p*-value), and the size of the points represents the VIP value. **(C)** KEGG pathway enrichment analysis for metabolites with VIP >1 and *p* (FDR) < 0.05. The horizontal axis represents the Rich Factor corresponding to each pathway, while the vertical axis displays the pathway names (sorted by *p*-value). The color of the dots reflects the magnitude of the *p*-value, with redder hues indicating more significant enrichment. The size of the dots represents the number of differential metabolites enriched in each pathway. OPLS-DA, orthogonal partial least squares-discriminant analysis; VIP, variable importance in projection; FC, fold change.

Orthogonal partial least squares-discriminant analysis (OPLS-DA) was applied to the data. The score plot showed separation between M0 and M4 samples (Figure 4A). A permutation test (*n* = 200) yielded a Q^2^ intercept *p*-value < 0.05, supporting the robustness of the model.

Using thresholds of |log_2_fold change| >0.584, VIP >1, and an unadjusted *p* < 0.05, 387 metabolites met criteria for altered relative abundance (220 down, 167 up). To reduce false discovery, we applied FDR correction (FDR < 0.05), resulting in 52 significantly altered metabolites (29 up-regulated, 23 down-regulated; Figure 4B). The top 20 metabolites by *p*-value, with their putative identifications, are listed (Supplementary Table S4).

These putatively identified metabolites were submitted to KEGG pathway enrichment analysis. Enriched pathways included “Lipid and atherosclerosis”, “Cholesterol metabolism”, “Insulin resistance”, and others (Figure 4C). Metabolites contributing to OPLS-DA separation are shown in a loading plot (Supplementary Figure S2). Given the exploratory nature of the analysis, these pathway associations are descriptive and not indicative of mechanistic causality.

## 4 Discussion

The present 16-week, randomized, double-blind, placebo-controlled trial investigated the effects of magnesium supplementation in a precisely defined high-risk population—older Chinese adults with both pre-diabetes and biochemical hypomagnesemia. The primary finding is that oral magnesium oxide supplementation significantly increased serum magnesium levels and led to a statistically significant reduction in fasting plasma glucose (FPG) compared to placebo. These findings, while modest in magnitude, provide targeted evidence supporting the role of magnesium repletion in individuals with confirmed deficiency. However, the interpretation of these results requires caution due to notable study limitations, including baseline imbalances in key metabolic parameters, the modest and isolated nature of the glycemic improvement, and the exploratory status of the mechanistic analyses.

Previous intervention studies in pre-diabetes have yielded mixed results, a discrepancy often attributed to heterogeneous baseline magnesium status among participants (16, 17). Our findings of significant improvements in the magnesium group align strongly with the trial by Guerrero-Romero et al. (11) which included 116 subjects with hypomagnesemia and newly diagnosed with Pre-DM. These subjects were randomized into two groups and received either 30 ml of 5% MgCl_2_ solution (equivalent to 382 mg of Mg) or an inert placebo solution once a day for 4 months. Conversely, Rezvan et al. (12) conducted a 12-week double-blind placebo-controlled randomized clinical trial to evaluate the effect of Mg oxide 250 mg/day supplementation on insulin resistance and cardiovascular indices in 86 Pre-DM patients which did not assess or select for baseline magnesium deficiency, found no significant glycemic benefit. This contrast underscores our core methodological premise: the therapeutic effect of magnesium supplementation on glucose metabolism is likely most pronounced and detectable in individuals who are truly deficient, highlighting the importance of targeted intervention strategies. Our high compliance rate (91.6%) and the stability of dietary magnesium intake (except for the provided supplement) further strengthen the internal validity of the causal inference.

However, the clinical relevance of the observed FPG reduction (~0.42 mmol/L in unadjusted analyses; See Results) is uncertain, as its impact on long-term diabetes risk remains unquantified in this study. The lack of significant effects on other integrated glycemic markers like HbA1c and glycated albumin may reflect the relatively short intervention duration, as these markers integrate glycemia over longer periods (18–21). The favorable but non-significant trend in HOMA-IR should be interpreted with caution, given the study's limited power for secondary endpoints and the significant baseline imbalance in this variable.

A critical methodological consideration is the presence of significant baseline differences between groups in insulin and HOMA-IR. Although we employed ANCOVA adjusted for baseline values to address this, residual confounding cannot be entirely ruled out. The similar improvements observed in some parameters (e.g., glycated albumin) in both groups may indicate the influence of non-specific factors, such as participation in a clinical trial (Hawthorne effect) or general lifestyle advice provided to all participants (22). Therefore, while the between-group difference in FPG is notable, the overall evidence for a robust, multi-faceted improvement in glucose metabolism from this single trial is limited.

Furthermore, the choice of magnesium formulation merits discussion. The modest reduction in FPG observed in this trial must be interpreted in the context of the magnesium formulation used. We selected magnesium oxide based on its high elemental magnesium content, cost-effectiveness, and widespread clinical availability, making it a pragmatic choice for a potential public health intervention (7). However, its relatively lower bioavailability compared to organic salts such as magnesium citrate or glycinate is a critical consideration. It is plausible, and even likely, that this formulation characteristic attenuated the physiological effect size. Had a higher-bioavailability form been administered, the same elemental magnesium dose might have resulted in a greater increment in intracellular or ionized magnesium levels, potentially leading to a more pronounced improvement in glycemic parameters. Therefore, the observed ~0.5 mmol/L reduction in FPG could represent a conservative estimate of the glucose-lowering potential of magnesium repletion in this population. This hypothesis directly informs future research: comparative efficacy trials are needed to determine whether using higher-bioavailability formulations yields stronger clinical effects, thereby clarifying the dose-response and formulation-response relationships for magnesium in pre-diabetes management.

The observed non-significant trend toward increased C-peptide in the magnesium group may suggest a potential favorable effect on beta-cell function or insulin clearance (23–26), warranting further investigation in larger trials. It should be noted that the effects of magnesium supplementation on C-peptide reported in previous studies have been inconsistent, with some showing a reduction in individuals with type 2 diabetes (27) or in overweight populations (23), and others reporting no significant between-group difference (28). These discrepancies are likely attributable to variations in study populations, magnesium formulations and dosages, intervention durations, and sample sizes. This further highlights the need for larger-scale validation specifically in populations with the phenotype targeted in this study—older adults with pre-diabetes and hypomagnesemia.

Contrary to some mechanistic hypotheses, we found no evidence that magnesium supplementation reduced systemic inflammation, as measured by hs-CRP and IL-6, in this population. This null finding is consistent with other researches (29, 30). It is plausible that the anti-inflammatory effects of magnesium are more pronounced in individuals with elevated baseline inflammation, which was not characteristic of our generally healthy, community-dwelling participants. This highlights the context-dependent nature of nutritional interventions and suggests that inflammation reduction may not be a primary mechanism of action for magnesium in all pre-diabetic populations.

The non-targeted metabolomics analysis suggested alterations in the serum metabolome after magnesium supplementation. While OPLS-DA indicated separation between timepoints and enrichment analysis associated putative metabolites with pathways such as “Insulin Resistance” and “Cholesterol metabolism”, these findings must be interpreted with caution. Key methodological limitations include the use of putative metabolite identifications without MS/MS confirmation, the exploratory nature of pathway enrichment analysis, and the descriptive, non-mechanistic character of the results. Although we applied FDR correction to reduce false discoveries, the results remain exploratory and should not be construed as evidence of causal or functional mechanisms. Instead, they generate specific hypotheses—particularly regarding magnesium's potential interaction with insulin resistance and lipid metabolism—for future validation through targeted metabolomics, stable isotope tracing, and functional studies.

This study possesses several notable strengths. First, it is a randomized, double-blind, placebo-controlled trial specifically targeting a well-defined high-risk phenotype—older adults with both pre-diabetes and biochemically confirmed hypomagnesemia—thereby addressing a significant evidence gap in this understudied population. Second, a key methodological strength lies in the systematic monitoring of dietary magnesium intake. By employing combined assessment tools and demonstrating stable background dietary magnesium levels throughout the intervention in both groups, we significantly reduced a major potential confounding factor, strengthening the causal attribution of the observed glycemic changes to the supplementation itself. Third, the integration of exploratory non-targeted metabolomics provides a novel, systemic perspective on the metabolic alterations associated with magnesium repletion, generating valuable hypotheses for future mechanistic research. These design features collectively enhance the internal validity of our findings.

Several important limitations should be considered when interpreting the findings of this study. First, the sample size was modest, and the trial was underpowered to detect significant effects for most secondary and exploratory outcomes. Second, the sole reliance on fasting plasma glucose (FPG) as the primary glycemic endpoint is a notable limitation. Given that dysglycemia in older adults often first manifests in the postprandial state, the lack of dynamic assessments—such as an oral glucose tolerance test (OGTT), postprandial glucose monitoring, or insulin secretion indices (e.g., the disposition index)—limits a comprehensive evaluation of glucose homeostasis and may have missed potential effects of magnesium supplementation on postprandial metabolism. Third, the use of magnesium oxide, a formulation with relatively lower bioavailability, may have constrained the magnitude of the observed physiological responses. Regarding the exploratory metabolomics analysis, several methodological constraints must be emphasized. Although we applied false discovery rate (FDR) correction to mitigate false positives, metabolite identifications remain putative in the absence of MS/MS confirmation with authenticated standards. Furthermore, the pathway enrichment analysis is descriptive in nature and should not be interpreted as evidence of causality or underlying biological mechanism. These findings are strictly hypothesis-generating and require validation through targeted analytical approaches. Finally, the 4-month intervention duration precludes any assessment of the long-term sustainability of the observed effects on FPG, as well as any potential impact on the progression to type 2 diabetes. Collectively, these limitations underscore the preliminary nature of the current findings and highlight critical avenues for future research, including larger, longer-duration trials that incorporate comprehensive glycemic phenotyping and validated biomarker assessments.

## 5 Conclusion

This pioneering randomized controlled trial confirms that magnesium supplementation successfully corrects hypomagnesemia in the targeted older population. Regarding glycemic outcomes, the intervention yielded a statistically significant but clinically modest reduction in fasting plasma glucose, without impacting other key parameters of glycemic control (HbA1c, insulin resistance, etc.). Therefore, this trial, which was not powered to detect changes in robust diabetes prevention markers, does not establish that magnesium supplementation provides a meaningful benefit for preventing diabetes based on the isolated FPG change observed over 16 weeks. Future large-scale, long-term studies are required to determine whether improving magnesium status translates into a clinically relevant reduction in diabetes risk.

## Data Availability

The raw data supporting the conclusions of this article will be made available by the authors, without undue reservation.
