# Supplementary material for: Oral magnesium supplementation improves glycemic control in older Chinese adults with pre-diabetes and hypomagnesemia: a randomized controlled trial
Source: Front Nutr. 2026 Feb 11;13:1765308. doi: 10.3389/fnut.2026.1765308 (PMC12932175; doi:10.3389/fnut.2026.1765308)
Supplement: Supplementary file 1 [file Table_1.docx]

Supplementary Material

# Sample preparation and extraction

- 1. Liquid samples class I

The sample stored at -80 °C refrigerator was thawed on ice and vortexed for 10 s. 50 μL of sample and 300 μL of extraction solution (ACN : Methanol = 1:4, V/V) containing internal standards were added into a 2 mL microcentrifugetube. The sample was vortexed for 3 min and then centrifuged at 12000 rpm for 10 min (4 °C). 200 μL of the supernatant was collected and placed in -20 °C for 30 min, and then centrifuged at 12000 rpm for 3 min (4 °C). A 180 μL aliquots of supernatant were transferred for LC-MS analysis.

- 1. HPLC Conditions

All samples were for two LC/MS methods. One aliquot was analyzed using positive ion conditions and was eluted from T3 column (Waters ACQUITY Premier HSS T3 Column 1.8 µm, 2.1 mm * 100 mm) using 0.1 % formic acid in water as solvent A and 0.1 % formic acid in acetonitrile as solvent B in the following gradient: 5 to 20 % in 2 min, increased to 60 % in the following 3 mins, increased to 99 % in 1 min and held for 1.5 min, then come back to 5 % mobile phase B within 0.1 min, held for 2.4 min. The analytical conditions were as follows, column temperature, 40 °C; flow rate, 0.4 mL/min; injection volume, 4 μL; Another aliquot was using negative ion conditions and was the same as the elution gradient of positive mode.

- 1. MS Conditions (QE)

All the methods alternated between full scan MS and data dependent MSn scans using dynamic exclusion. MS analyses were carried out using electrospray ionization in the positive ion mode and negative ion mode using full scan analysis over m/z 75-1000 at 35000 resolution. Additional MS settings are: ion spray voltage, 3.5 KV or 3.2 KV in positive or negative modes, respevtively; Sheath gas (Arb), 30; Aux gas, 5; Ion transfer tube temperature, 320 °C; Vaporizer temperature, 300 °C; Collision energy, 30,40,50 V; Signal Intensity Threshold, 1*e6 cps; Top N vs Top speed, 10; Exclusion duration, 3s.

# Analytical Methods

## PCA

Unsupervised PCA (principal component analysis) was performed by statistics function prcomp within R (www.r-project.org). The data was unit variance scaled before unsupervised PCA.

## Hierarchical Cluster Analysis and Pearson Correlation Coefficients

The HCA (hierarchical cluster analysis) results of samples and metabolites were presented as heatmaps with dendrograms, while pearson correlation coefficients (PCC) between samples were caculated by the cor function in R and presented as only heatmaps. Both HCA and PCC were carried out by R package ComplexHeatmap. For HCA, normalized signal intensities of metabolites (unit variance scaling) are visualized as a color spectrum.

## Differential metabolites selected

For two-group analysis, differential metabolites were determined by VIP (VIP > 1) and P-value (P-value < 0.05, Student’s t test). VIP values were extracted from OPLS-DA result, which also contain score plots and permutation plots, was generated using R package MetaboAnalystR. The data was log transform (log$\text{​}_{\text{2}}$) and mean centering before OPLS-DA. In order to avoid overfitting, a permutation test (200 permutations) was performed.

## KEGG annotation and enrichment analysis

Identified metabolites were annotated using KEGG Compound database (<http://www.kegg.jp/kegg/compound/>), annotated metabolites were then mapped to KEGG Pathway database (<http://www.kegg.jp/kegg/pathway.html>).

## Software and Analytical Methods List

Table: Software and Specifications for Data Analysis

| Analysis | Software | Version | Data Processing Method |
| --- | --- | --- | --- |
| KNN | R (impute) | 1.56.0 | default parameters |
| PCA | R (base package) | 4.1.2 | UV (unit variance scaling) |
| Heatmap | R (ComplexHeatmap) | 2.9.4 | UV (unit variance scaling) |
| Pearson Correlation Coefficients | R (base package) | 4.1.2 | - |
| Sample Correlation Plot | R (corrplot) | 0.92 | - |
| OPLS-DA | R (MetaboAnalystR) | 1.0.1 | Log₂ Transformation + Mean Centering |
| Radar Chart | R (fmsb) | 0.7.1 | - |
| Chord Diagram | R (igraph; ggraph) | 1.2.11; 2.0.5 | - |
| Correlation Network Graph | R (igraph) | 1.2.11 | - |
| K-Means Clustering Plot | R (base package) | 4.1.2 | UV (unit variance scaling) |

Two primary data scaling/normalization methods were applied during the metabolomics analysis:

(1) UV (Unit Variance Scaling) / Z-score Normalization / Auto Scaling

This method standardizes data based on the mean and standard deviation of the original data. The processed data conform to a standard normal distribution, with a mean of 0 and a standard deviation of 1.

Formula: $\text{x}^{\text{′}}\text{=}\frac{\text{x}\text{−}\text{μ}}{\text{σ}}$

where $\text{μ}$ is the mean and $\text{σ}$ is the standard deviation.

(2) Mean Centering / Zero-Centering(Ctr)

Calculation：The mean of the variable is subtracted from the original data.

Formula: $\text{x}^{\text{′}}\text{=}\text{x}\text{−}\text{μ}$

where µ is the mean.

# Supplementary Figures and Tables

## Supplementary Figures


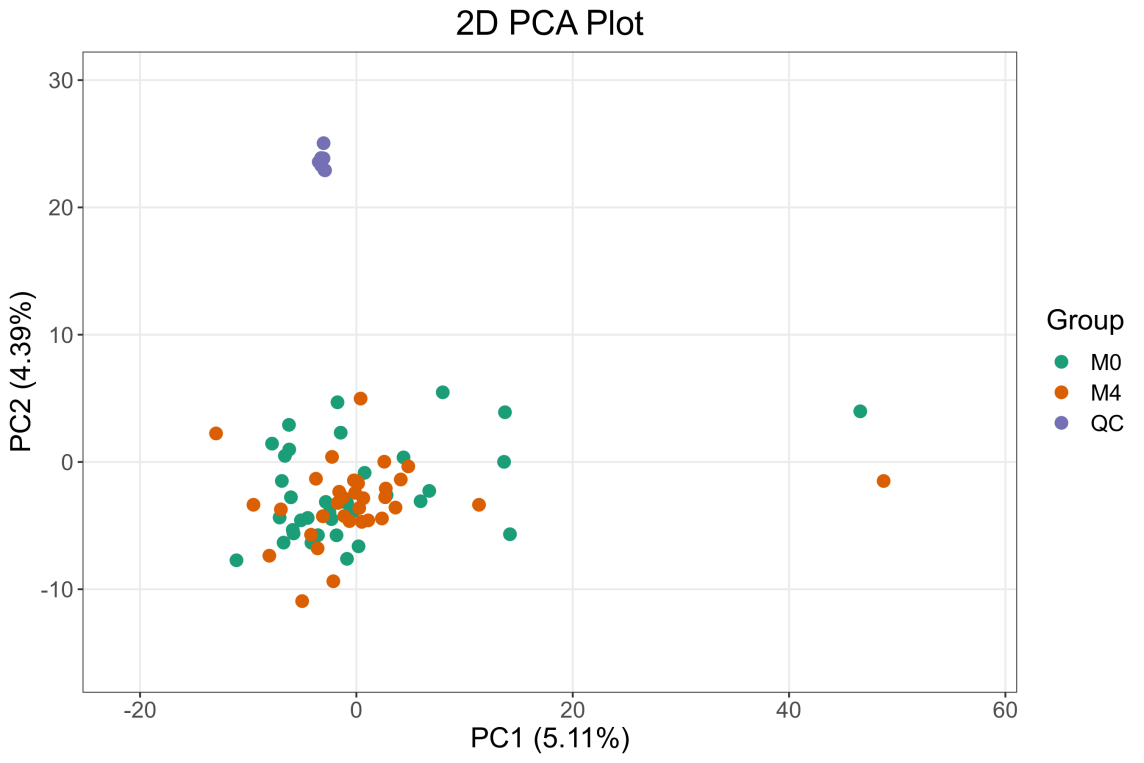


**Supplementary Figure S1.** **Principal component analysis (PCA) plot of all serum samples including quality controls (QCs) demonstrates analytical stability.**

This 2D PCA plot, based on the untargeted metabolomics data, includes both experimental samples (M0, baseline; M4, after 4-month intervention) and pooled QC samples injected throughout the analytical sequence. The tight clustering of QC samples (green triangles) in the PCA space, distinctly separated from the experimental groups (M0 and M4), indicates high reproducibility and minimal technical variation during the entire LC-MS/MS run, thereby validating the robustness of the acquired metabolomics data.


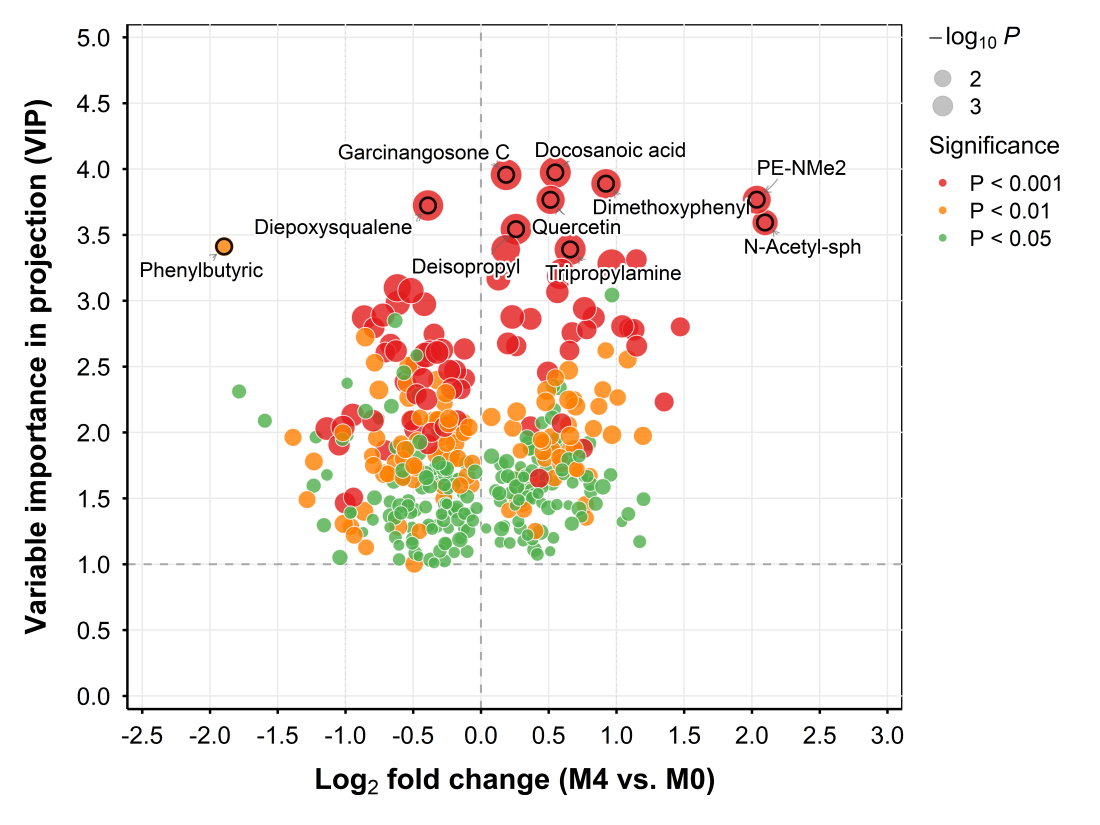


**Supplementary Figure S2. VIP score plot of serum metabolites altered by magnesium supplementation (M4 vs. M0).**

Points represent metabolites colored by significance: red (P < 0.001), orange (P < 0.01), green (P < 0.05), gray (not significant). Point size corresponds to −log₁₀(P-value). Black circles highlight top 10 metabolites with VIP > 1 and P < 0.05. Dashed lines: VIP = 1.0 (horizontal) and log₂FC = 0 (vertical). All 387 detected metabolites showed significant alterations (P < 0.05), with 167 upregulated and 220 downregulated.

## Supplementary Tables

| **Supplementary Table S1. Inclusion and Exclusion Criteria for the Study** | |
| --- | --- |
| **Category** | **Criteria** |
| **Inclusion Criteria** | 1. Age and Residence: Aged 60–80 years, community-dwelling residents, able and willing to complete scheduled follow-ups. |
|  | 2. Glycemic Status - Prediabetes: Meeting any one of the following criteria based on American Diabetes Association (ADA) standards: |
|  | • Impaired Fasting Glucose (IFG): 5.6 ≤ FPG < 7.0 mmol/L. |
|  | • Impaired Glucose Tolerance (IGT): 7.8 ≤ 2hPG < 11.1 mmol/L. |
|  | • Combined State (IFG+IGT): Meeting both IFG and IGT criteria above. |
|  | • Glycated Hemoglobin (HbA1c): 5.7% ≤ HbA1c < 6.5%. |
|  | 3. Serum Magnesium Level: Serum magnesium concentration ≤0.80 mmol/L. |
| **Exclusion Criteria** | 1. History of Diabetes Mellitus: Diagnosis of diabetes mellitus. |
|  | 2. Relevant Medication Use: Current use of medications known to affect glucose or mineral metabolism (e.g., diuretics, insulin, oral hypoglycemic agents). |
|  | 3. Mineral Supplement Intake: Intake of magnesium, calcium, or other mineral supplements within the preceding three months. |
|  | 4. Major Chronic Diseases: Significant renal impairment (estimated glomerular filtration rate < 45 mL/min/1.73 m²) or other major chronic diseases (e.g., severe hepatic dysfunction, heart failure). |
|  | 5. Gastrointestinal Conditions: Severe gastrointestinal disorders or history of gastrointestinal surgery that could affect absorption. |
|  | 6. Allergy or Intolerance: Known allergy or intolerance to the intervention components. |
|  | 7. Compliance and Follow-up: Inability or unwillingness to complete the study follow-up. |

| **Supplementary Table S2. Intervention Effects on Biochemical Outcomes** | | | | | | | | | |
| --- | --- | --- | --- | --- | --- | --- | --- | --- | --- |
| **Variable** | **Placebo_M0** | **Placebo_M4** | **Placebo_Δ** | **Mg_M0** | **Mg_M4** | **Mg_Δ** | **Δ_p-value** | **ANCOVA**  **p-value** | **Effect size (95% CI)** |
| **Mg** | 0.78 ± 0.05 | 0.82 ± 0.06 | 0.040 ± 0.059 | 0.78 ± 0.05 | 0.89 ± 0.07 | 0.112 ± 0.076 | <0.001 | <0.001 | 0.056 (0.028 to 0.085) |
| **FPG** | 6.17 ± 0.68 | 6.40 ± 0.90 | 0.225 ± 0.587 | 6.69 ± 1.84 | 6.27 ± 1.31 | -0.424 ± 0.979 | 0.001 | 0.003 | -0.497 (-0.818 to -0.176) |
| **HbA1c** | 5.79 ± 0.42 | 5.84 ± 0.43 | 0.055 ± 0.289 | 5.99 ± 0.72 | 5.88 ± 0.53 | -0.114 ± 0.523 | 0.096 | 0.203 | -0.109 (-0.274 to 0.057) |
| **Insulin** | 12.00 ± 7.70 | 12.14 ± 7.99 | 0.143 ± 4.484 | 16.63 ± 10.69 | 15.02 ± 9.78 | -1.613 ± 6.541 | 0.191 | 0.581 | -0.739 (-3.346 to 1.869) |
| **HOMA_IR** | 3.34 ± 2.23 | 3.58 ± 2.82 | 0.242 ± 1.662 | 5.35 ± 5.07 | 4.47 ± 3.68 | -0.879 ± 2.736 | 0.041 | 0.296 | -0.495 (-1.417 to 0.426) |
| **C_peptide** | 2.64 ± 1.07 | 2.53 ± 1.21 | -0.112 ± 0.429 | 2.65 ± 1.07 | 2.95 ± 1.17 | 0.291 ± 1.248 | 0.074 | 0.512 | 0.126 (-0.248 to 0.499) |
| **GA** | 14.89 ± 2.76 | 13.45 ± 1.77 | -1.439 ± 2.030 | 14.77 ± 3.34 | 13.38 ± 1.53 | -1.392 ± 2.635 | 0.934 | 0.983 | 0.007 (-0.611 to 0.625) |
| **IL6** | 3.34 ± 3.70 | 2.51 ± 1.15 | -0.827 ± 3.523 | 2.78 ± 3.21 | 3.17 ± 3.35 | 0.396 ± 1.990 | 0.079 | 0.116 | 0.835 (-0.192 to 1.863) |
| **hsCRP** | 2.12 ± 2.72 | 1.80 ± 2.44 | -0.319 ± 2.888 | 1.75 ± 1.63 | 1.41 ± 1.48 | -0.339 ± 1.936 | 0.972 | 0.457 | -0.361 (-1.307 to 0.584) |
| Δ represents the change from baseline; ANCOVA analysis adjusted for baseline values, sex, insulin, and HOMA-IR. | | | | | | | | | |

| **Supplementary Table S3. Comparison of change scores (4-month minus baseline) between Mg and placebo groups** | | | | | | | | | |
| --- | --- | --- | --- | --- | --- | --- | --- | --- | --- |
| **Variable** | **Placebo_Change** | **Mg_Change** | **Mean_Difference** | **p_value** | **FDR_adjusted_p** | **CI_95** | **Cohen_d** | **Statistical**  **_Power** | **Significance** |
| Mg | 0.040 ± 0.059 | 0.112 ± 0.076 | 0.073 | <0.001 | <0.001 | 0.040 to 0.105 | 1.06 | 99.2% | * |
| FPG | 0.225 ± 0.587 | -0.424 ± 0.979 | -0.649 | 0.001 | 0.006 | -1.032 to -0.267 | -0.80 | 91.0% | * |
| HbA1c | 0.055 ± 0.289 | -0.114 ± 0.523 | -0.169 | 0.096 | 0.145 | -0.369 to 0.031 | -0.40 | 37.6% | ns |
| Insulin | 0.14 ± 4.48 | -1.61 ± 6.54 | -1.756 | 0.191 | 0.245 | -4.410 to 0.898 | -0.31 | 25.1% | ns |
| HOMA_IR | 0.24 ± 1.66 | -0.88 ± 2.74 | -1.122 | 0.041 | 0.122 | -2.194 to -0.050 | -0.49 | 53.1% | ns |
| C_peptide | -0.11 ± 0.43 | 0.29 ± 1.25 | 0.403 | 0.074 | 0.141 | -0.041 to 0.847 | 0.43 | 42.6% | ns |
| GA | -1.439 ± 2.030 | -1.392 ± 2.635 | 0.046 | 0.934 | 0.972 | -1.066 to 1.159 | 0.02 | 5.1% | ns |
| IL6 | -0.827 ± 3.523 | 0.396 ± 1.990 | 1.223 | 0.079 | 0.141 | -0.144 to 2.590 | 0.43 | 42.4% | ns |
| hsCRP | -0.319 ± 2.888 | -0.339 ± 1.936 | -0.021 | 0.972 | 0.972 | -1.192 to 1.150 | -0.01 | 5.0% | ns |

| **Supplementary Table S4. The top 20 significantly altered metabolites** | | | | | |
| --- | --- | --- | --- | --- | --- |
| **Compounds** | **VIP** | **P_value** | **Log2FC** | **Type** | **ShortName** |
| **Docosanoic acid, 1-[[[(2-aminoethoxy)hydroxyphosphinyl]oxy]methyl]-2-[(1-oxoeicosyl)oxy]ethyl ester, (R)-** | 3.974 | 2.22e-11 | 0.549 | up | Docosanoic acid...(R) |
| **Garcimangosone C** | 3.956 | 1.19e-10 | 0.185 | up | Garcimangosone C |
| **2-(3,5-dimethoxyphenyl)-5-hydroxy-7-methoxy-6-(3-methylbut-2-en-1-yl)-3,4-dihydro-2H-1-benzopyran-4-one** | 3.888 | 2.37e-09 | 0.923 | up | 2 (3...one |
| **PE-NMe2(18:0/24:1(15Z))** | 3.768 | 2.56e-08 | 2.035 | up | PE-NMe2(18:0/24:1(15Z)) |
| **Quercetin** | 3.765 | 1.41e-08 | 0.514 | up | Quercetin |
| **Diepoxysqualene** | 3.723 | 2.50e-10 | -0.391 | down | Diepoxysqualene |
| **N-acetylsphinganine** | 3.593 | 9.93e-07 | 2.097 | up | N-acetylsphinganine |
| **Deisopropylhydroxyatrazine** | 3.543 | 1.60e-10 | 0.259 | up | Deisopropylhydroxyat... |
| **2-Phenylbutyric acid** | 3.412 | 1.23e-03 | -1.896 | down | 2-Phenylbutyric acid |
| **Tripropylamine** | 3.389 | 3.59e-11 | 0.659 | up | Tripropylamine |
| **Heptylmalonic acid** | 3.389 | 7.54e-09 | 0.183 | up | Heptylmalonic acid |
| **4-Formyl-2-hydroxybenzoic acid** | 3.312 | 2.04e-04 | 1.147 | up | 4 Formyl...acid |
| **[(2S)-2-[(Z)-hexadec-9-enoyl]oxy-3-pentadecanoyloxypropyl] (6Z,9Z,12Z)-octadeca-6,9,12-trienoate** | 3.282 | 9.83e-09 | 0.965 | up | [(2S) 2...trienoate |
| **TG(14:0/18:1(11Z)/15:0)** | 3.225 | 2.03e-06 | 0.593 | up | TG(14:0/18:1(11Z)/15:0) |
| **1-(11Z,14Z-eicosadienoyl)-2-pentadecanoyl-glycero-3-phosphocholine** | 3.185 | 8.99e-07 | 0.581 | up | 1 (11Z...phosphocholine |
| **10-Hydroxy-2-decenoic acid** | 3.17 | 4.85e-06 | 0.129 | up | 10 Hydroxy...acid |
| **Pseudolaric acid B** | 3.099 | 6.54e-08 | -0.617 | down | Pseudolaric acid B |
| **Diethyl disulfide** | 3.077 | 8.58e-07 | -0.516 | down | Diethyl disulfide |
| **Deoxypeganine** | 3.067 | 3.38e-05 | 0.564 | up | Deoxypeganine |
| **9-Oxo-nonanoic acid** | 3.044 | 1.36e-02 | 0.967 | up | 9-Oxo-nonanoic acid |
